# White matter maturation profiles through early childhood predict general cognitive ability

**DOI:** 10.1007/s00429-014-0947-x

**Published:** 2014-11-29

**Authors:** Sean C. L. Deoni, Jonathan O’Muircheartaigh, Jed T. Elison, Lindsay Walker, Ellen Doernberg, Nicole Waskiewicz, Holly Dirks, Irene Piryatinsky, Doug C. Dean, N. L. Jumbe

**Affiliations:** 1 Advanced Baby Imaging Lab, Brown University School of Engineering, 184 Hope Street, Box D, Providence, RI 02912 USA; 2 Department of Neuroimaging, King’s College London, Institute of Psychiatry, London, UK; 3 Institute of Child Development, University of Minnesota, Minneapolis, USA; 4 The Bill and Melinda Gates Foundation, Seattle, USA

**Keywords:** Brain development, Myelination, Cognitive maturation, White matter growth, Neurodevelopment

## Abstract

Infancy and early childhood are periods of rapid brain development, during which brain structure and function mature alongside evolving cognitive ability. An important neurodevelopmental process during this postnatal period is the maturation of the myelinated white matter, which facilitates rapid communication across neural systems and networks. Though prior brain imaging studies in children (4 years of age and above), adolescents, and adults have consistently linked white matter development with cognitive maturation and intelligence, few studies have examined how these processes are related throughout early development (birth to 4 years of age). Here, we show that the profile of white matter myelination across the first 5 years of life is strongly and specifically related to cognitive ability. Using a longitudinal design, coupled with advanced magnetic resonance imaging, we demonstrate that children with above-average ability show differential trajectories of myelin development compared to average and below average ability children, even when controlling for socioeconomic status, gestation, and birth weight. Specifically, higher ability children exhibit slower but more prolonged early development, resulting in overall increased myelin measures by ~3 years of age. These results provide new insight into the early neuroanatomical correlates of cognitive ability, and suggest an early period of prolonged maturation with associated protracted white matter plasticity may result in strengthened neural networks that can better support later development. Further, these results reinforce the necessity of a longitudinal perspective in investigating typical or suspected atypical cognitive maturation.

## Background

Early neurodevelopment is a complex process, during which the brain structurally matures alongside, and in response to, dramatic changes in cognitive ability and behavioral functions. Throughout infancy and early childhood, myelination, synaptogenesis, axonal pruning, and other neurodevelopmental processes respond to genetic and environmental cues to create efficient brain networks that subserve motor, visual, behavioral, cognitive and emotive functioning. Of long-standing interest is the relationship between brain development and cognitive ability (Casey et al. 2005; Durston and Casey 2006). Previous cross-sectional studies have examined differences in cognition and intelligence with respect to brain size (Reiss et al. 1996), global and regional white and gray matter volume (Gur et al. 1999; Wilke et al. 2003; Narr et al. 2007), sub-cortical gray matter volume (Andreasen et al. 1993; Frangou et al. 2004), and, more recently, cortical thickness (Shaw et al. 2006) and measures of white matter microstructure (Nagy et al. 2004; Schmithorst et al. 2005; Colom et al. 2010; Tang et al. 2010).

Longitudinal investigations have provided improved insight into the relationships between brain development and the evolution of cognitive ability, revealing dynamic relationships between intelligence and brain structure that vary with age (Giedd et al. 1999; Nagy et al. 2004; Shaw et al. 2006; Ramsden et al. 2011). For example, the cortex of highly intelligent children has been found to follow a very different trajectory of development than in less intelligent children; with a prolonged period of rapid cortical thickening in late childhood followed by accelerated thinning in adolescence (Shaw et al. 2006). Mirroring these gray matter and cortical changes, white matter (WM) micro-structural development has also been linked to intellectual ability with significant age interaction (Nagy et al. 2004; Fields 2010; Jernigan et al. 2012; Borghesani et al. 2013; Short et al. 2013). Moreover, deviations from normal development have been associated with behavioral and cognitive disorders, including autism spectrum disorders (ASD), in which children with ASD appear to have accelerated early brain growth over the first 2 years of life followed by more protracted or stagnant growth through the remainder of childhood (Courchesne 2004; Courchesne et al. 2007).

While the myelinated WM plays an important role in cognitive processing by facilitating high-speed and high-fidelity communication across brain networks (Fields 2010; Zatorre et al. 2012), few studies have sought to relate WM maturation to the emergence of cognitive ability and intelligence during infancy (Short et al. 2013; O’Muircheartaigh et al. 2014). Rather, prior imaging studies have focused almost exclusively on older children (4 years of age and above), after the bulk of WM development has occurred. This is an important gap since brain and cognitive development is most rapid between birth and 4 years of age (Johnson 2001), and this time period is believed to correspond with the emergence on several developmental disorders, including ASD (Xiao et al. 2014), ADHD (Halperin et al. 2012), and other developmental and intellectual disorders (Mazzocco and Thompson 2005).

Here we sought to explore the relationship between general cognitive ability and a specific process underlying WM development (i.e., myelination) in a longitudinal study of children from 3 months to 5 years of age. We longitudinally characterized WM myelination, using a multicomponent relaxation approach to calculate the myelin water fraction (MWF) (Deoni et al. 2008), in a large group (*n* = 257, 109 female) of typically developing children. Cognitive ability was assessed using the Mullen Scales of Early Learning (Mullen 1995), which provides a standardized general score, the Early Learning Composite (ELC), that reflects the average of fine motor function, visual processing, and receptive and expressive language skills. Verbal and non-verbal ability were also independently examined using the Verbal and Non-Verbal Developmental Quotients (VDQ and NVDQ) (Short et al. 2013) of the Mullen Scales. We hypothesized that ELC, VDQ and NVDQ would be significantly associated with MWF cross-sectionally in young children, and that longitudinal trajectories of MWF vs. age would differ significantly between children stratified by general cognitive ability.

## Materials and methods

### Participants

257 healthy and typically developing children (109 female), between 98 and 1,814 days (approximately 3 months through 5 years of age), corrected to a 40-week gestation, recruited from the local Rhode Island community, were included in this study. This cohort of children were selected from a larger population of children our group has imaged and assessed as part of on-going studies of normal and atypical development (O’Muircheartaigh et al. 2014; Dean et al. 2014b, d). Inclusion criteria included: (1) healthy singleton birth between 37 and 42 weeks gestation; (2) uncomplicated pregnancy and delivery; (3) APGAR scores >8; (4) no reported abnormalities on fetal ultrasound; (5) no reported neurological history in the child; (6) no reported psychiatric or learning disability history in the child or first degree relatives. Full demographic information is provided in Table 1. Inclusion criteria were confirmed during phone interview prior to enrollment, and again through infant and family medical history information obtained as part of the study. Extensive infant, parent, and sibling medical and family history questionnaires were used to provide additional information regarding each child’s neurological and psychiatric history; maternal and paternal education levels; maternal prenatal and postnatal health, substance use, and breastfeeding practices; gestation duration; and birth weight. Maternal SES was determined using the Hollingshead four factor index (Hollingshead 1975). Written informed consent was obtained from each child’s parents or legal guardian and the study was performed with approval from the Brown University Internal Review Board.

**Table 1:** Participant demographic information

Full sample	
Gender	
Male (*n*)	148
Female (*n*)	109
Racial background	
Caucasian (*n*)	179
African American (*n*)	25
Asian (*n*)	6
Mixed race (*n*)	37
Mean age (days)	784 ± 521
Age range (days)	98–1,814
Mean gestation (weeks)	39.1 ± 3.2
Mean birth weight (lbs)	7.4 ± 1.1
Mean maternal SES	5.53 ± 1.54
Infants	
Gender	
Male (*n*)	20
Female (*n*)	18
Racial background	
Caucasian (*n*)	17
African American (*n*)	1
Asian (*n*)	2
Mixed race (*n*)	8
Mean age (days)	234 ± 44
Age range (days)	98–363
Mean gestation (weeks)	39.3 ± 1.1
Mean birth weight (lbs)	7.4 ± 1.1
Mean maternal SES	5.3 ± 1.8
Mean ELC	93.8 ± 16.6
Toddlers	
Gender	
Male (*n*)	43
Female (*n*)	43
Racial background	
Caucasian (*n*)	64
African American (*n*)	10
Asian (*n*)	0
Mixed race (*n*)	12
Mean age (days)	482 ± 103
Age range (days)	365–725
Mean gestation (weeks)	39.4 ± 1.3
Mean birth weight (lbs)	7.5 ± 0.95
Mean maternal SES	5.3 ± 1.98
Mean ELC	94.6 ± 15.1
Young children	
Gender	
Male (*n*)	56
Female (*n*)	48
Racial background	
Caucasian (*n*)	69
African American (*n*)	14
Asian (*n*)	4
Mixed race (*n*)	17
Mean age (days)	1,296 ± 325
Age range (days)	737–1,814
Mean gestation (weeks)	38.9 ± 1.8
Mean birth weight (lbs)	7.3 ± 1.2
Mean maternal SES	5.83 ± 1.03
Mean ELC	97.7 ± 18.1

All 257 enrolled children were scanned and cognitively assessed at least once. A subset of 126 children received additional longitudinal scans. In general, children under 2 years of age were imaged and cognitively assessed at 6-month intervals; and children older than 2 were scanned and assessed yearly. Of the 126 children included in the longitudinal component, all were imaged twice; 39 were imaged at least three times; 15 were imaged at least 4 times; and 4 were imaged 5 times. The mean inter-scan interval was approximately 9 months (280 days). On the basis of their cognitive scores, this subset was divided into one of three groups: above average (*n* = 38); average (*n* = 54); and below average (*n* = 34). There were no significant group differences in male:female ratio or racial composition (evaluated using *χ* ^2^ tests), mean age, gestation duration, birth weight, or maternal SES (all evaluated using two-tailed unpaired *t* tests). The longitudinal group demographics are provided in Table 2. The number of total scans acquired in each group was: above average = 85; average = 126; and below average = 76, each covering the age range from ~98 to ~1,814 days of age.

**Table 2:** Longitudinal participant demographics

			*p* value
Below average			
Gender			
Male	23	*χ* ^2^ = 2.19	0.33
Female	11		
Racial background			
Caucasian	22	*χ* ^2^ = 4.25	0.64
African American	0		
Asian	8		
Mixed race	4		
Cognitive ability			
Mean ELC	79.8 ± 5.4	***F*** **=** **199**	**<0.00001**
Mean NVDQ	3.03 ± 0.46	***F*** **=** **28**	**<0.00001**
Mean VDQ	2.65 ± 0.56	***F*** **=** **39**	**<0.00001**
Mean age (days)	868 ± 98	*F* = 1.01	0.36
Age range (days)	98–1,808		
Mean gestation (weeks)	39.7 ± 1.5	*F* = 1.06	0.35
Mean birth weight (lbs)	7.2 ± 1.5	*F* = 0.11	0.9
Mean maternal SES	5.23 ± 1.8	*F* = 2.04	0.14
Average			
Gender			
Male	28		
Female	26		
Racial background			
Caucasian	38		
African American	2		
Asian	7		
Mixed race	7		
Cognitive ability			
Mean ELC	98.8 ± 6.5		
Mean NVDQ	3.35 ± 0.43		
Mean VDQ	3.21 ± 0.55		
Mean age (days)	888 ± 98		
Age range (days)	101–1,814		
Mean gestation (weeks)	39.4 ± 1.3		
Mean birth weight (lbs)	7.3 ± 1.1		
Mean maternal SES	5.7 ± 1.3		
Above average			
Gender			
Male	22		
Female	16		
Racial background			
Caucasian	27		
African American	2		
Asian	4		
Mixed race	5		
Cognitive ability			
Mean ELC	121.1 ± 5.2		
Mean NVDQ	3.8 ± 0.51		
Mean VDQ	3.8 ± 0.57		
Mean age (days)	898 ± 76		
Age range (days)	99–1,807		
Mean gestation (weeks)	39.8 ± 1.4		
Mean birth weight (lbs)	7.2 ± 1.0		
Mean maternal SES	5.97 ± 1.73		

### MRI imaging and MWF calculation

All children were scanned during natural, *non*-*sedated* sleep, or, if tolerated by the older children, while watching a favorite movie. To obtain voxel-wise MWF measures, the mcDESPOT multicomponent relaxometry technique was used (Deoni et al. 2008, 2013). Age-optimized mcDESPOT protocols (Table 3) comprise series of spoiled gradient recalled echo (SPGR) images and fully balanced steady-state free precession (bSSFP) images acquired over a range of flip angles (Deoni et al. 2012). Inversion-prepared (IR-)SPGR data were also acquired to correct for transmit magnetic field (i.e., B_1_ field) inhomogeneities (Deoni 2011); and the bSSFP data were acquired with two different phase-cycling patterns to allow correction for main magnetic field (i.e., B_0_ field) inhomogeneities (Deoni 2011). A constant voxel dimension of (1.8 × 1.8 × 1.8) mm^3^ was used for all children, with the field of view and imaging matrix adjusted depending on age. To minimize acoustic noise, the maximum imaging gradient slew rates and peak values were reduced, and passive measures, including a sound-insulating bore liner, MiniMuff ear pads, and sound-attenuating ear protectors were used (Dean et al. 2014a).

**Table 3:** Age-optimized mcDESPOT imaging protocols

Age group	3–9 months	9–16 months	16–28 months	28–60 months
Acquisition time (min)	18:22	18:42	21:38	0 h 24 m 20 s
Field of view (cm)	14 × 14 × 13	17 × 17 × 14.4	18 × 18 × 15	20 × 20 × 15
Image matrix	80 × 80 × 76	96 × 96 × 80	104 × 104 × 84	112 × 112 × 84
SPGR TE/TR (ms)	5.8 ms/12 ms	5.9 ms/12 ms	5.4 ms/12 ms	5.2 ms/11 ms
SPGR flip angles (°)	2, 3, 4, 5, 7, 9, 11, 14	2, 3, 4, 5, 7, 9, 11, 14	2, 3, 4, 5, 7, 9, 11, 14	2, 3, 4, 5, 7, 9, 12, 16
SPGR bandwidth (Hz/pixel)	350	350	350	350
IR-SPGR TI/TE/TR (ms)	(600, 950) ms/5.8 ms/12 ms	(600, 900) ms/5.9 ms/12 ms	(500, 850) ms/5.4 ms/12 ms	(500, 800) ms/5.2 ms/11 ms
IR-SPGR flip angle (°)	5	5	5	5
IR-SPGR image matrix	80 × 80 × 38	96 × 96 × 40	108 × 104 × 42	112 × 112 × 42
bSSFP TE/TR (ms)	5 ms/10 ms	5.1 ms/10.2 ms	5 ms/10 ms	4.4 ms/9.8 ms
bSSFP flip angles (°)	9, 14, 20, 27, 34, 41, 56, 70	9, 14, 20, 27, 34, 41, 56, 70	9, 14, 20, 27, 34, 41, 56, 70	9, 14, 20, 27, 34, 41, 56, 70
bSSFP bandwidth (Hz/pixel)	350	350	350	350

Following acquisition, each child’s raw SPGR, IR-SPGR and bSSFP images were linearly co-registered to account for subtle intra-scan motion (Jenkinson et al. 2002), and non-brain signal was removed (Smith 2002). B_0_ and B_1_ field calibration maps were then calculated, followed by MWF map calculation through the iterative fitting of a three-pool tissue model using a constrained fitting approach that provides stable estimates (Deoni and Kolind 2014).

### Image registration

Following MWF map calculation, each child’s map was non-linearly co-registered to a common standardized space. Described in more detail previously (Deoni et al. 2012), this involves a two-step procedure. First, using the high flip angle T_1_-weighted SPGR image acquired as part of mcDESPOT, the MWF maps were non-linearly co-registered to an age-specific template using the ANTS package (Avants et al. 2011; Deoni et al. 2012). A final (precomputed) transformation from each age template to MNI space was then applied (Deoni et al. 2012). Once all MWF maps were transformed to standard space, they were smoothed with a 4-mm full-width-at-half-maximum 3D Gaussian kernel applied within a white and gray matter mask.

For the longitudinal data, a modified registration approach was used (Reuter et al. 2012). Independently registering longitudinal measurements to a common template may introduce subtle inconsistencies when not accounting for the repeated regularity of subject-specific measurements (Dean et al. 2014c). To minimize this potential source of variability, an individual T_1_-weighted template was first created for each longitudinal participant using the subject’s high flip angle SPGR images. The non-linear transformation from this individual template to the age-specific template was then calculated. The individual’s MWF maps were then transformed to MNI space by first transforming to their individual template, to the age-specific template, and then to MNI space, in a single interpolation step (Dean et al. 2014d).

### Cognitive assessments

Within 1 week of a successful MRI, all children were assessed using the Mullen Scales of Early Learning (Mullen 1995), which provides age-normalized scores for fine and gross motor control, visual reception, and expressive and receptive language for children up to 5 years, 9 months of age. In addition to the individual domain scores, the Mullen Scales provide an overall composite score (the Early Learning Composite, ELC, expressed as a standard score with mean = 100 and standard deviation of 15) derived from the sum of the fine motor, visual reception, and expressive and receptive language age-normalized *T*-scores. Verbal and non-verbal ability may also be examined independently using the Verbal Developmental Quotient (VDQ, derived from the expressive and receptive language scores) and the Non-Verbal Developmental Quotient (NVDQ, derived from the fine motor and visual reception scores) (Rogers et al. 2003).

In addition to the Mullen’s, assessments of abnormal behavior, including the Modified Checklist for Autism in Toddlers (M-CHAT) (Chlebowski et al. 2013) and the Communication and Symbolic Behavior Scales Developmental Profile (CSBS-DP) (Wetherby et al. 2002), were used to identify children who may have suspected developmental or learning delay. No children who screened at-risk using these broad assessment tools were included in this study.

### Statistical testing

#### Cross-sectional analysis

**Fig. 1:**
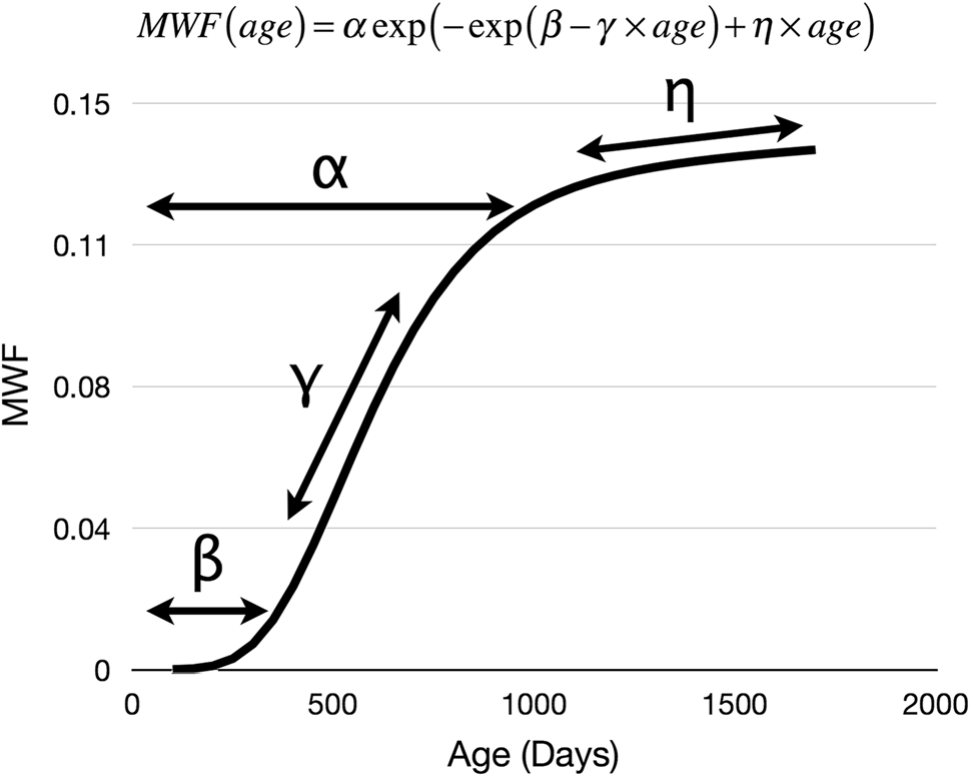
Example of modified Gompertz growth model defined by four parameters that reflect an initial period of slow growth (*β*), initial growth rate (*γ*), a period of transition from fast to slower growth (*α*), and a secondary growth rate (*η*)

With all data aligned in a common space, we investigated the relationship between MWF and general cognitive ability (ELC) in the full cohort of 257 children using a general linear model (GLM) that modeled ELC and its interaction with age. To account for the non-linear dependence of MWF on age, we first fit a modified Gompertz growth model (Dean et al. 2014d) to the data at each voxel. A sigmoidal shape, this curve is defined by four parameters as shown in Eq. (1),

1 $${\text{MWF}}\;({\text{age}}) = \alpha \;\exp ( - \exp (\beta - \gamma \times {\text{age}}) + \eta \times {\text{age}}),$$

which correspond to the initial onset of growth (*β*), initial growth rate (*γ*), a second reflection where growth begins to slow considerably (*α*), and a final linear growth rate (*η*) as shown in Fig. 1.

Using a bootstrap resampling approach (Efron and Tibshirani 1994), we predicted the population MWF mean and variance at each child’s age and used this to Z-normalize each child’s MWF value. Child age was further included as a covariate of no interest within the GLM to further ensure age effects were accounted for. Birthweight, gestation, and maternal SES were also included in the GLM. To account for multiple comparisons, a cluster-based approach (threshold-free cluster-enhancement, TFCE) was used (Smith and Nichols 2009). Resultant cluster-corrected *p* value maps were then superimposed on the MNI space T_1_-weighted template and a threshold of *p* < 0.05 applied. The testing of ELC × age interaction was included to investigate the temporal stability of the relationship over the age period.

To investigate differences in the MWF vs. ELC relationship as a broad function of age, we divided our population into three continuous age groups: infants (3–12 months, *n* = 38); toddlers (12–24 months, *n* = 86); and young children (2–5 years, *n* = 104) matched for gender (*χ* ^2^ = 0.28, *p* = 0.87) and racial composition (*χ* ^2^ = 10.1, *p* = 0.12), and mean ELC (*F*_3,228_ = 1.16, *p* = 0.32) (Table 1). In these three age-cohorts, we performed the same analysis as above.

Relationships between MWF and VDQ and NVDQ separately were investigated in a similar manner in the full cohort, as well as in the three age-subgroups, using a GLM that included age, birthweight, gestation, and SES as covariates of no interest. NVDQ was included as a covariate in the VDQ analysis and vice versa to examine these relationships independently and specifically.

#### Longitudinal analysis

We used the longitudinal data to formally investigate potential differences in early childhood brain development trajectories in children stratified by cognitive ability. On the basis of the mean ELC measure across at least 2 assessments, 126 children were classified as either Above Average (mean ELC > 115); Average (85 < mean ELC < 115); or Below Average (ELC < 85), respecting mean ± one standard deviation of the normalized ELC T-scores. Group demographics are provided in Table 2. Children were classified on the basis of *mean* ELC to reduce potential group mis-assignment due to natural variability with age (Ramsden et al. 2011). Final group characteristics were: Above Average: *n* = 38, mean ELC = 121.1 ± 5.2, total scan # = 82; Average: *n* = 54, mean ELC = 98.8 ± 6.5, total scan # = 126; and Below Average: *n* = 34, mean ELC = 79.8 ± 5.4, total scan # = 76. There were no significant group differences in gender (*χ* ^2^ = 2.19, *p* = 0.33) or racial composition (*χ* ^2^ = 4.25, *p* = 0.64); age (*p* = 0.36); gestation duration (*p* = 0.35); birthweight (*p* = 0.9); or maternal SES (*p* = 0.14) (Table 2).

Using non-linear mixed-effects regression (Lindstrom and Bates 1990; Wu and Zhang 2006), we calculated mean Gompertz curves for each group for whole-brain white matter (MW), as well as frontal, temporal, occipital, parietal, and cerebellar WM, and the body, genu, and splenium of the corpus callosum. For each region, growth models were calculated independently for each ability group, and a single model was fit to the combined data. An *F* test was used to confirm the data supported three independent models. For confirmed areas, we then compared the *α*, *β*, *γ*, and *η* parameters of the growth model (Fig. 1) between each group using an analysis of variance and an ad hoc Tukey HSD test. Significance was defined as *p* < 0.0013 (*p* < 0.05 corrected for the 36 comparisons using Bonferroni correction). For easy visual comparison, continuous MWF growth trajectories for each ability group were generated using the calculated and plotted.

## Results

**Fig. 2:**
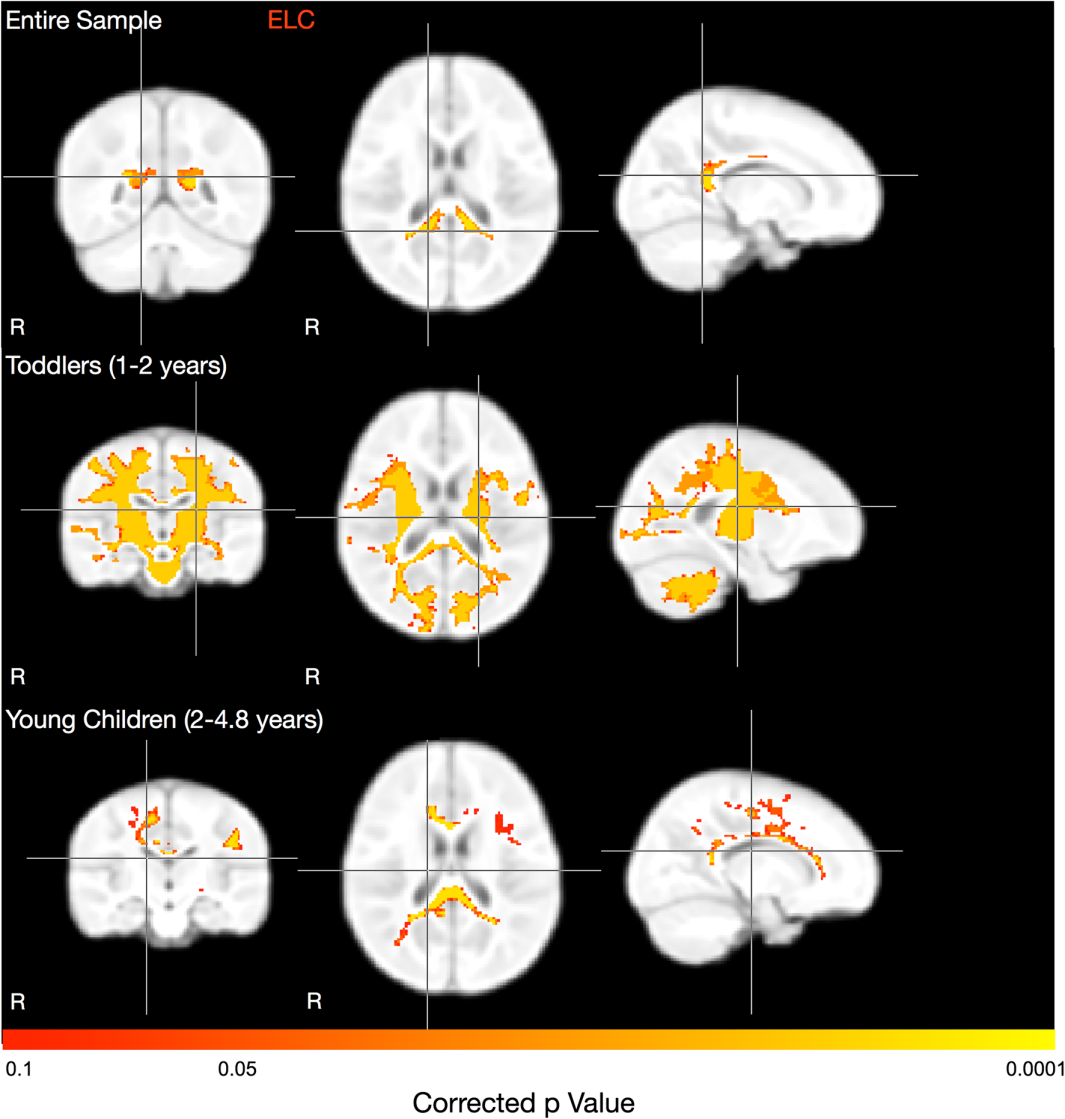
Correlations between ELC and MWF. Across the full cohort we found a significant (*p* < 0.05 corrected) positive correlation between overall cognitive ability and MWF in the splenium of the corpus callosum. In the individual age groups, we found significant positive correlations were identified in the body and splenium of the corpus callosum; bilateral internal capsule, corticospinal tracts and primary motor and somatosensory cortices, optic radiations, superior longitudinal fasciculus, posterior cingulate, visual and auditory cortex, and cerebellar white matter in toddlers. In young children, significant positive correlations were found in throughout the corpus callosum; left Wernicke’s area and primary somatosensory cortex; and right premotor cortex and anterior cingulate

**Fig. 3:**
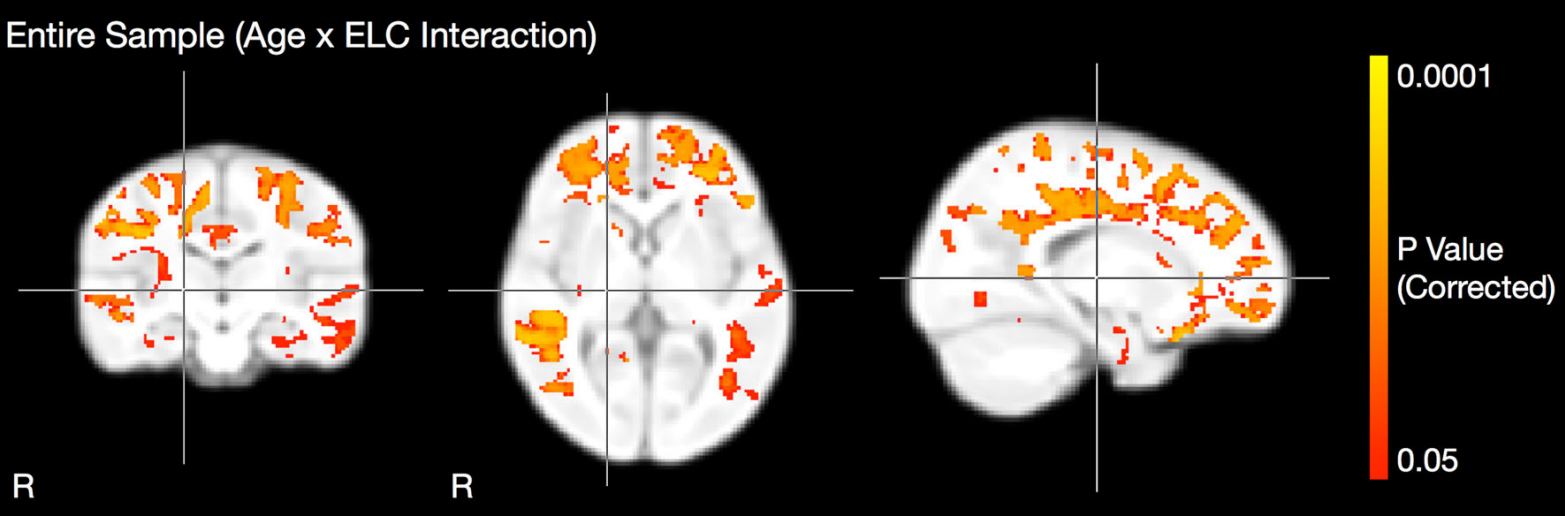
Across the full cohort of children, we also examined age × ELC interaction within our GLM framework. Brain regions exhibiting significant interaction are shown and include frontal, parietal, and temporal lobe WM, as well as splenium and body of the corpus callosum

The relationship between ELC and MWF was first investigated cross-sectionally in the full 257 child cohort, with each child contributing a single MWF dataset. Results revealed a significant positive relationship (*p* < 0.05, cluster corrected for multiple comparisons; Smith and Nichols 2009) in the posterior portion (splenium) of the corpus callosum (Fig. 2, top row). Significant age × ELC interactions were found more globally throughout the brain, including frontal, parietal, and temporal lobe WM, as well as splenium and body of the corpus callosum (Fig. 3).

Within the three continuous age groups: infants (3–12 months, *n* = 38); toddlers (12–24 months, *n* = 86); and young children (2–5 years, *n* = 104) we found marked age-related differences (Fig. 2, second and third rows). In infants, a trend towards a positive relationship (*p* < 0.1, corrected) was found in the right inferior parietal lobe, and anterior superior longitudinal fasciculus; and bilateral Broca’s area, and premotor cortex (not shown in Fig. 2). In toddlers, significant positive associations were identified in the body and splenium of the corpus callosum; bilateral internal capsule, corticospinal tracts, primary motor, somatosensory, visual and auditory cortices, optic radiations, forceps, superior longitudinal fasisculus, and cerebellar WM. Finally, in young children, significant positive correlations were found throughout the corpus callosum, and primary somatosensory cortex; and right premotor cortex and anterior cingulum. Cumulatively, these results suggest an early and variable period of functional onset (infancy), a period of widespread associations and development (toddlers) and, finally, a period of structure–function consolidation.

**Fig. 4:**
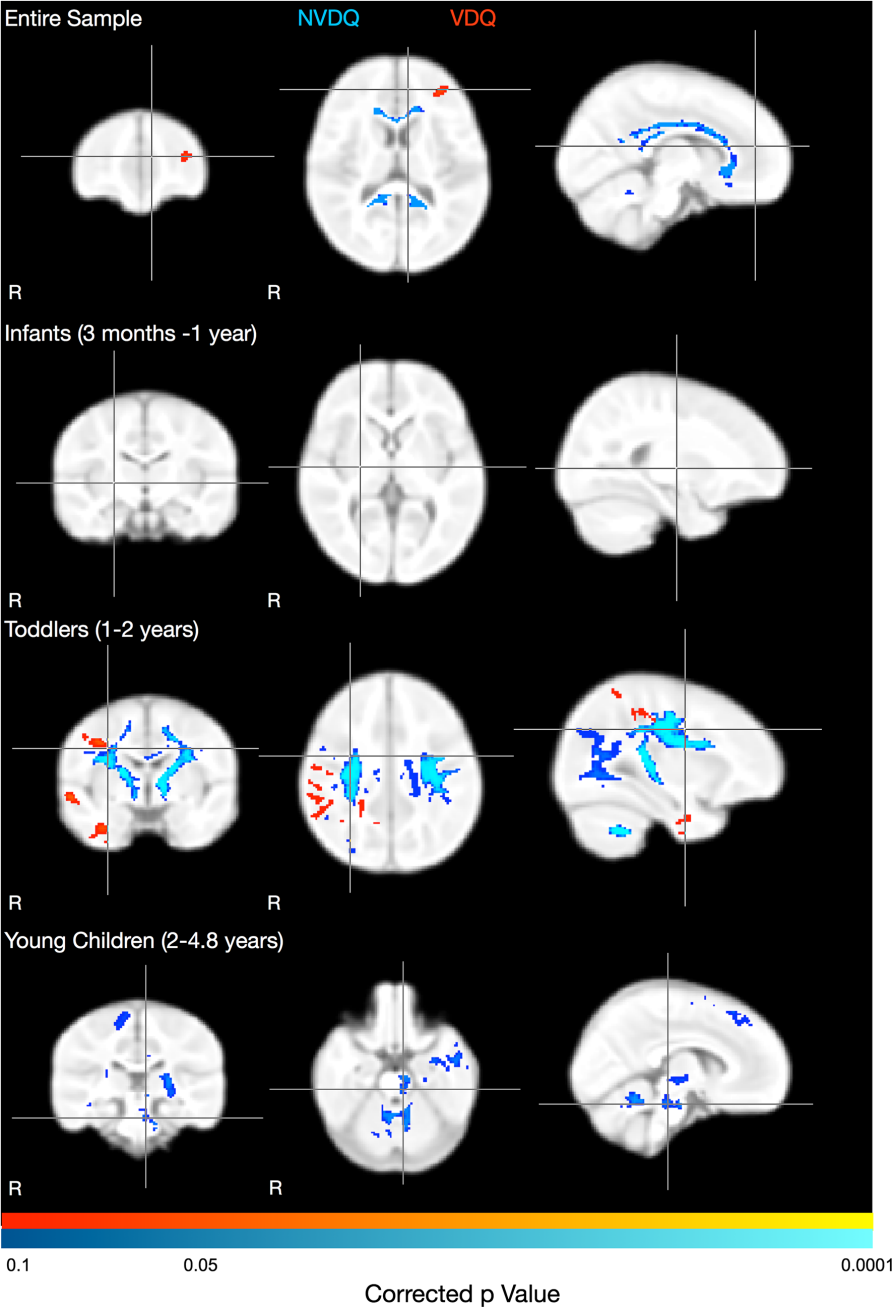
Correlations between MWF and VDQ (*red-yellow*) and NVDQ (*light* to *dark blue*). Significant positive associations between MWF and NVDQ were identified throughout the corpus callosum and left cerebellum in the full cohort. A trend towards a significant relationship between MWF and VDQ was also identified in left Broca’s area (*p* < 0.09). In the individual age groups, no associations were identified in the infant group. In toddlers, MWF and NVDQ were significantly associated in bilateral corticospinal tracts, cerebellum, and premotor and primary motor cortices; and a trend towards a significant (*p* < 0.10, corrected) MWF and VDQ association was found in right temporal lobe and superior longitudinal fasciculus. In young children, a significant MWF and NVDQ association was found in bilateral cerebellum, left temporal lobe, left internal capsule and right premotor and primary motor cortices

A similar trend of anatomical consolidation was observed with respect to VDQ and NVDQ (Fig. 4). Across the full cohort (Fig. 4, top row), significant positive relationships between MWF and NVDQ were found throughout the corpus callosum and cerebellar white matter, while the association between MWF and VDQ was restricted to left Broca’s area (not significant after correction for multiple comparisons, *p* < 0.09 corrected). This suggests the observed MWF vs. ELC relationships are primarily, but not completely, driven by NVDQ associations, and that there is specialization within discrete neural circuits. Examining the infant, toddler, and young child cohorts separately, no significant associations between MWF and NVDQ or VDQ were observed in the infants. Within the toddlers, significant positive relationships between MWF and NVDQ were found diffusively throughout the motor network, including cerebellum, internal capsule, corpus callosum, and motor cortices, as well as occipital lobe WM. A trend towards significance (*p* < 0.1 corrected) between MWF and VDQ was found in right temporal WM postcentral gyrus, and supramarginal gyrus. In young children, significant associations between MWF and NVDQ were observed in cerebellar and brainstem WM, left temporal lobe, left internal capsule and left motor cortices. No significant MWF vs. VDQ associations were identified in young children.

**Fig. 5:**
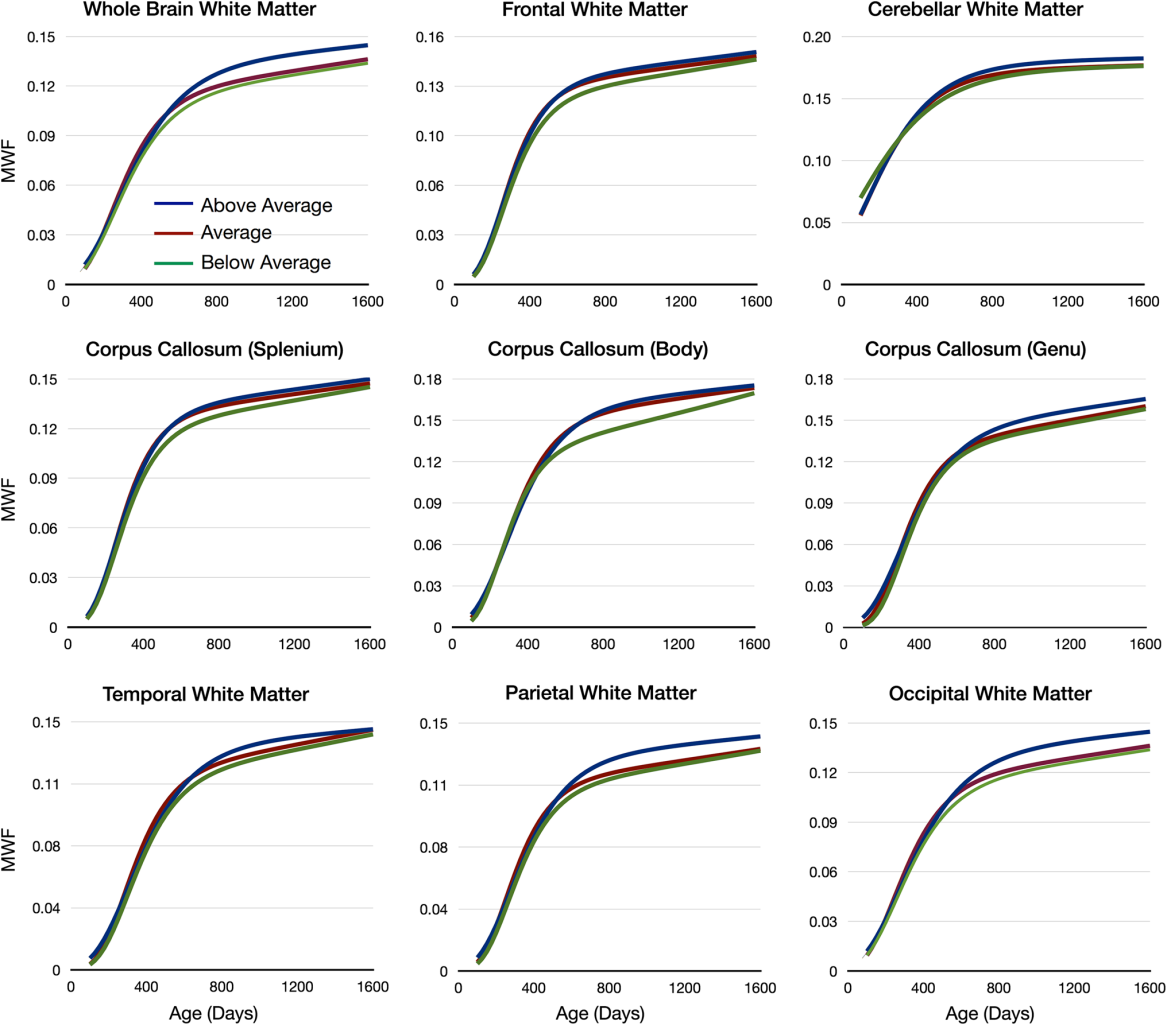
Reconstructed continuous mean Gompertz growth curves for each brain region investigated. In all cases, a developmental trend with above average ELC (*blue lines*) > average ELC (*red lines*) > below average ELC (*green lines*)

We hypothesized that longitudinal MWF development would differ between children stratified by cognitive ability. Exploring this formally using non-linear mixed-effects modeling in a subset of 126 children with repeated scans and assessments, we found above average, average and below average children had significantly different trajectories of MWF development in each of the brain regions investigated (Fig. 5, Table 4).

**Table 4:** Comparisons of individual growth model parameters between ability level groups

Brain region	Model term	Above average	Average	Below average
Global WM	*α*	0.13 (0.007)^†,‡^	0.11 (0.003)	0.11 (0.006)
	*β*	1.33 (0.09)^†,‡^	1.50 (0.09)	1.43 (0.16)
	*γ*	0.005 (0.0005)^†,‡^	0.007 (0.0004)^ℑ^	0.006 (0.0007)
	*η*	0.0005 (0.00004)^†,‡^	0.0001 (0.00002)	0.0001 (0.00004)
Body CC	*α*	0.15 (0.009)^†,‡^	0.14 (0.005)^ℑ^	0.12 (0.007)
	*β*	1.56 (0.12)^†,‡^	1.73 (0.12)^ℑ^	1.96 (0.32)
	*γ*	0.006 (0.0006)^†,‡^	0.007 (0.0005)^ℑ^	0.009 (0.0001)
	*η*	0.0007 (0.00004)^†,‡^	0.0001 (0.00003)^ℑ^	0.002 (0.00005)
Genu CC	*α*	0.14 (0.011)^†,‡^	0.12 (0.005)	0.12 (0.006)
	*β*	1.63 (0.22)^†,‡^	2.05 (0.18)^ℑ^	2.22 (0.19)
	*γ*	0.006 (0.0009)^†,‡^	0.008 (0.0007)	0.008 (0.0007)
	*η*	0.0001 (0.00006)^†,‡^	0.0002 (0.00003)	0.0002 (0.00003)
Splenium CC	*α*	0.13 (0.006)^†,‡^	0.12 (0.004)^ℑ^	0.12 (0.006)
	*β*	1.77 (0.15)^†^	1.91 (0.11)	1.86 (0.21)
	*γ*	0.008 (0.0007)^†^	0.008 (0.0005)^ℑ^	0.008 (0.0009)
	*η*	0.0001 (0.00004)^‡^	0.0001 (0.00003)^ℑ^	0.0001 (0.00004)
Cerebellar WM	*α*	ANOVA, *p* > 0.05		
	*β*	0.56 (0.08)^‡^	0.57 (0.04)^ℑ^	0.024 (0.13)
	*γ*	0.005 (0.0006)	0.0005 (0.0004)^ℑ^	0.004 (0.0009)
	*η*	0.0001 (0.00004)^‡^	0.0002 (0.00001)	0.0002 (0.000005)
Frontal WM	*α*	0.12 (0.008)^†,‡^	0.10 (0.005)^ℑ^	0.098 (0.006)
	*β*	1.53 (0.12)^†,‡^	1.82 (0.15)	1.86 (0.21)
	*γ*	0.005 (0.0005)^†,‡^	0.007 (0.0006)	0.007 (0.0008)
	*η*	0.0008 (0.00005)^†,‡^	0.0002 (0.00004)^ℑ^	0.0002 (0.00004)
Temporal WM	*α*	0.13 (0.009)^†,‡^	0.11 (0.005)^ℑ^	0.11 (0.006)
	*β*	1.56 (0.12)^†,‡^	1.91 (0.12)	1.90 (0.19)
	*γ*	0.005 (0.0005)^†,‡^	0.0007 (0.0005)^ℑ^	0.007 (0.0007)
	*η*	0.0005 (0.00005)^†,‡^	0.0001 (0.00003)^ℑ^	0.0002 (0.00004)
Parietal WM	*α*	0.12 (0.007)^†,‡^	0.11 (0.004)^ℑ^	0.10 (0.005)
	*β*	1.54 (0.12)^†,‡^	1.80 (0.11)	1.82 (0.19)
	*γ*	0.006 (0.0006)^†,‡^	0.0078 (0.0005)	0.007 (0.0008)
	*η*	0.0005 (0.00004)^†,‡^	0.0001 (0.00003)^ℑ^	0.0001 (0.00003)
Occipital WM	*α*	0.15 (0.009)^†,‡^	0.13 (0.004)	0.13 (0.007)
	*β*	0.99 (0.08)^†^	1.12 (0.09)^ℑ^	0.97 (0.13)
	*γ*	0.005 (0.0005)^†,‡^	0.007 (0.0005)^ℑ^	0.006 (0.0007)
	*η*	0.0004 (0.00004)^†,‡^	0.0001 (0.00003)	0.0001 (0.00003)

^†^Significant difference between the Above average and Average groups; ^‡^ significant difference between the Above average and Below average groups; and ^ℑ^ between the Average and Below average groups. All significance is defined as *p* < 0.0013

**Fig. 6:**
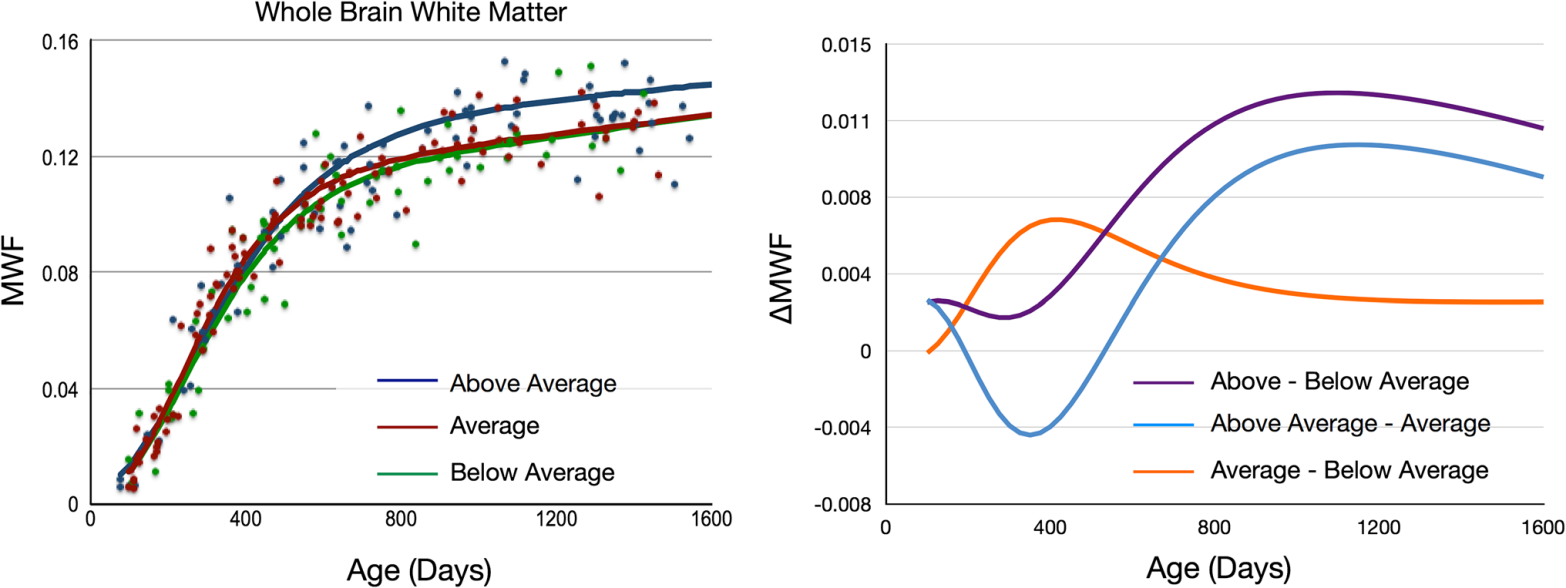
Comparison of whole-brain white matter MWF development. Reconstructed continuous growth models for mean whole-brain white matter calculated using the longitudinal data from the above average (*blue*), average (*red*), and below average (*green*) ELC children. Individual data points represent the mean values calculated from each individual child’s data. The results show a trend seen in all other investigated brain regions, with overall development in the above-average children > average > below average. Differences in development are more clearly visualized by examining the ΔMWF curves, which show an early delay in myelination onset and prolonged myelination in the above-average children, resulting in an overall increase in MWF in later childhood

In all regions, the above-average group displayed greater MWF values by 3 years of age compared to the average and below-average groups (Fig. 5). Looking specifically at the whole-brain white matter, plots of group MWF difference (ΔMWF) (Fig. 6) highlight the different developmental trajectories associated with each group. These plots suggest the above-average children have initially slower, but prolonged, period of WM maturation through the first 2 years of life. These trends can be seen more directly through comparison of the Gompertz growth model parameters. The above-average group consistently exhibits a longer initial lag period (denoted by *β*, Fig. 1) and slower initial growth rate (*γ* term), but longer growth phase (*α* term) and faster secondary growth rate (*η* term) than either the average or below-average groups. This trajectory yields a maximal MWF difference by ~3 years of age. In contrast, the average group showed increased early development relative to below average children with these groups appearing to normalize with age.

## Discussion

Early neurodevelopment is punctuated by inter-related developmental processes, including myelination, synaptogenesis, and synaptic pruning, which yield highly optimized and efficient brain networks and systems. With respect to myelination, this process begins before birth (at approximately 26–28 weeks gestation) and proceeds rapidly over the first 2 years of life before continuing more slowly throughout childhood, adolescence and early adulthood (Davison and Dobbing 1966; Holland et al. 1986). Synaptogenesis follows a similarly protracted timeline, with peak cortical synaptic density occurring between 3.5 and 4 years of age (depending on cortical region), after which synaptic refinement and pruning results in a significant decline in synapse number (Huttenlocher and Dabholkar 1997). Each of these processes respond to activity, experience, and learning, as well as genetic cues, throughout the lifespan. Using brain imaging methods, indirect measures of myelination (through MWF or WM volumetric imaging) and synapse density (cortical thickness or gray matter density), may be obtained and related to changes in cognition or ability (Giedd et al. 1999; Nagy et al. 2004; Shaw et al. 2006; Ramsden et al. 2011).

To assess general cognitive ability, we utilized the Mullen Scales of Early Learning. Consisting of five scales (fine and gross motor, visual reception, and expressive and receptive language), the Mullen’s provides a comprehensive measure of cognitive functioning in infants and young children up to 68 months of age. Unfortunately, to our knowledge, no study has associated domain or composite scores obtained from the Mullen’s with common intelligence scales used in older children. However, in a study of hearing impaired children, the visual reception sub-test of the Mullen’s was found to predict the Full IQ score of the Leiter International Performance Scales-Revised (Sullivan 1998; Caudle et al. 2012). Further, analysis of ‘g’ loadings found that the Mullen expressive and receptive language scales had the highest g loadings for school-aged children in the Wechsler Intelligence Scale for Children, 3rd Ed. (Wechsler 1991; Mullen 1995), which lend support for the validity of the Mullen ELC as a measure of general cognitive ability.

In this work, we examined the relationships between white matter myelination and cognitive ability, and their evolution with age over the first 5 years of childhood. Though few studies have examined WM microstructure or volume throughout this early neurodevelopmental period, our finding of increased WM MWF in older, high ability children, coincides with prior reports of WM microstructure change associated with improved working memory score, processing speed, and verbal intelligence quotient in older children (Nagy et al. 2004; Fields 2010; Zatorre et al. 2012). Our findings generally intimate that higher cognitive ability is associated with slower initial development over the first year of life, followed by a prolonged period of rapid maturation between 1 and 2 years of age. Considering the overlap in the timelines of synaptogenesis, synaptic pruning and myelination, these results may suggest that the early period of slowed myelination coincides with increased synaptogenesis, with the subsequent prolonged period of rapid development coinciding, or associated, with a prolonged period of synaptic pruning. However, this association is speculative since we do not have corresponding measures of cortical thickness across these early life stages.

Across the full cohort of children, we found a significant association between MWF and general cognitive ability (ELC) localized to the corpus callosum. This result aligns with prior studies of older children, which have consistently associated callosal morphology and structure with cognitive ability (Luders et al. 2011). Abnormalities of the corpus callosum have also been implicated in a variety of developmental disorders, including ASD (Vidal et al. 2006; Just et al. 2007). Maturation of the corpus callosum occurs across childhood and adolescence, culminating at the age at which we achieve our maximum speed in cognitive processing and skills (Schulz and Curnow 1988; Pujol et al. 1993). Myelination of the corpus callosum does not occur all across all segments simultaneously, rather, in order from the genu to body to splenium (Deoni et al. 2011). Volumetric studies of callosal development also demonstrate a continual increase in callosum size through the third decade of life, suggesting it is part of the highest order and latest maturing brain network (Pujol et al. 1993). Given the importance and involvement of the corpus callosum in a wide variety of cognitive functions, and its protracted development, it is unsurprising that this was the only brain area we found to be significant associated with ELC in children at all ages.

Expectantly, our results demonstrate the relationships between cognitive ability and brain structure are not static, but evolve with age. Our results suggest an early and variable period of functional onset (infancy), during which relationships are subtle. This is followed by a period of widespread associations and development (toddlers), during which general cognitive ability is diffusely associated with brain structure, but with specialization to discrete brain networks and circuits. For example, non-verbal function is associated with white matter regions throughout motor systems, including cerebellum, corticospinal tracts, and motor cortices; and verbal functioning is associated with more language-oriented regions, including the supramarginal gyrus and temporal lobe (Fig. 4). The reason for the observed localization of verbal associations in right hemisphere areas, as opposed to left hemisphere, is not clear. While the left hemisphere is more traditionally associated with language skills in adults (Gaillard et al. 2003), lesion and more recent TMS studies suggest the right supramarginal gyrus plays an important role in phonological processing (Hartwigsen et al. 2010). Finally, we observe a period of structure–function consolidation (childhood), during which associations become more localized and specific.

Overall, we noted distinct developmental trajectories were associated with cognitive ability and outcome. While prior studies have examined WM development with respect to cognition and learning (Nagy et al. 2004; Fields 2008), this is the first study to examine this critical process throughout early childhood. Further, though prior longitudinal studies of white matter maturation have been performed (Lebel et al. 2008; Lebel and Beaulieu 2011), the relationship between observed trajectories and developing cognition has been unclear. In studies examining cortical development in older children (Giedd et al. 1999; Shaw et al. 2006), highly intelligent children were found to have thinner cortices than lesser abled children at 5 years of age. Our results may suggest that improved network efficiency, potentially provided by increased WM myelination in these children, may allow the cortex to mature more gradually, and with increased plasticity, in high ability children. Prior investigations of WM volume and cortical thickness have shown cortical thinning is spatially and temporally concomitant with brain volume growth, with the authors suggesting thinning may be driven by increased myelination of neural fibers in the lower cortical layers (Sowell et al. 2004). A current model of development posits that more gradual rates of development yield increased cortical volumes, particularly in areas important to intelligence (Clancy et al. 2000). This slower development may afford greater environmental interaction and fine-tuning of neural systems and, thus, a symbiotic relationship may exist between WM and cortical development (Van Essen 1997; Leppänen and Nelson 2008).

Although we identified reduced myelin development across the first year of life in the above-average children compared with the average and below-average ability children, we did not observe a linear trend. That is, the difference between the above-average and average children was greater than the difference between the above average and below average children. Indeed, the average ability children were found to develop more quickly across this period than those with below average ELC. This result was found in all investigated brain regions except the cerebellum. The reason for this trend is not immediately clear. One like explanation is that by examining only myelination, we are seeing only one piece of a complex picture. For example, we do not have measures of synapse density. Further, our measure of MWF provides only a voxel-wise measure related to myelin content, it does not inform on the thickness or number of myelin lamina on each neuronal axon. It may be that below-average children have fewer axons with greater myelin thickness than average children, which results in more rigid networks. To further investigate this hypothesis, measures of the myelin thickness or axon diameter (*g*-ratio) are needed (Campbell et al. 2014).

While the measure of myelin water fraction has a lengthy history in white matter disorders, such as multiple sclerosis (Laule et al. 2008; Mackay et al. 2009; Kolind et al. 2012) and amyotrophic lateral sclerosis (Kolind et al. 2013). In addition, the MWF imaging technique utilized herein, mcDESPOT, differs from some of the more conventional spin-echo T_2_-based approaches (MacKay et al. 1994). Histological verification of mcDESPOT has, to date, been limited to qualitative histological comparisons in the Shaking Pup model of dysmyelination (Hurley et al. 2010) and indirectly through comparison with the known histological time-course of myelination in human infants (Deoni et al. 2011, 2012), and demyelination studies in MS (Kolind et al. 2012; Kitzler et al. 2012). Thus, the specificity of mcDESPOT MWF measures as solely reflecting myelin may be questioned. Additional effects, such as magnetization transfer may also influence mcDESPOT values. However, animal model and in vivo results garnered so far give confidence that if not specific to myelin, mcDESPOT provides novel information regarding white matter microstructure, and offers differing, perhaps enhanced, sensitivity to myelin changes (Deoni et al. 2012). While the reliability of mcDESPOT MWF measures is also important (Lankford and Does 2013), we have recently shown that mcDESPOT returns robust and reliable estimates (Deoni and Kolind 2014).
